# 3D imaging of the brain morphology and connectivity defects in a model of psychiatric disorders: MAP6-KO mice

**DOI:** 10.1038/s41598-017-10544-2

**Published:** 2017-09-04

**Authors:** Ulysse Gimenez, Benoit Boulan, Franck Mauconduit, Fanny Taurel, Maxime Leclercq, Eric Denarier, Jacques Brocard, Sylvie Gory-Fauré, Annie Andrieux, Hana Lahrech, Jean Christophe Deloulme

**Affiliations:** 1 INSERM, U1205, BrainTech Lab, F-38000 Grenoble, France; 2 Univ. Grenoble Alpes, F-38000 Grenoble, France; 3 INSERM, U1216, Grenoble Institut des Neurosciences, F-38000 Grenoble, France; 4 Commissariat à l’Energie Atomique, BIG-GPC, F-38000 Grenoble, France

## Abstract

In the central nervous system, microtubule-associated protein 6 (MAP6) is expressed at high levels and is crucial for cognitive abilities. The large spectrum of social and cognitive impairments observed in MAP6-KO mice are reminiscent of the symptoms observed in psychiatric diseases, such as schizophrenia, and respond positively to long-term treatment with antipsychotics. MAP6-KO mice have therefore been proposed to be a useful animal model for these diseases. Here, we explored the brain anatomy in MAP6-KO mice using high spatial resolution 3D MRI, including a volumetric T_1w_ method to image brain structures, and Diffusion Tensor Imaging (DTI) for white matter fiber tractography. 3D DTI imaging of neuronal tracts was validated by comparing results to optical images of cleared brains. Changes to brain architecture included reduced volume of the cerebellum and the thalamus and altered size, integrity and spatial orientation of some neuronal tracks such as the anterior commissure, the mammillary tract, the corpus callosum, the corticospinal tract, the fasciculus retroflexus and the fornix. Our results provide information on the neuroanatomical defects behind the neurological phenotype displayed in the MAP6-KO mice model and especially highlight a severe damage of the corticospinal tract with defasciculation at the location of the pontine nuclei.

## Introduction

Microtubule effectors regulate microtubule organization and play key roles during brain development. In neurons, microtubule effectors contribute to a variety of neuronal functions involved in brain wiring such as migration and neuronal polarization, elongation and axonal guidance^1^. Mutations of genes encoding microtubule effectors including Microtubule-Associated Proteins (MAPs) such as Disrupted-In Schizophrenia1 (DISC-1), the lissencephaly protein and doublecortin, cause dysfunctional neuronal connectivity and a variety of structural brain abnormalities^2, 3^. These mutations lead to a large spectrum of neurological and psychiatric disorders, including schizophrenia, bipolarity, lissencephaly, double cortex syndrome or microcephaly^4–8^. In accordance with the involvement of MAPs in brain disorders, when microtubule-associated protein 6 is deleted in mice (MAP6-KO), severe behavioral disorders are observed, such as locomotor hyperactivity, severe social withdrawal, and cognitive deficits^9–14^. These animals also exhibit abnormalities in glutamatergic and dopaminergic neurotransmission, deficits associated with neuronal and synaptic plasticity and impaired sensorimotor gating^10, 13, 15–18^. Altogether, these effects are reminiscent of the clinical features observed in psychiatric diseases, in particular schizophrenia for the cognitive defects. Interestingly, some of the behavioral and biological defects of MAP6-KO can be partially alleviated by long-term treatment with neuroleptics or antidepressants^9, 13, 19, 20^. The MAP6-KO is therefore a relevant model for the study of schizophrenia.

In our previous study, and in studies by other groups, some changes to brain anatomy and connectivity were described in MAP6-KO mice, such as a reduced brain volume, depleted serotoninergic axonal projections, severe hypoplasia of long-distance projecting axon tracts, and an absence of the post-commissural fornix^14, 17, 21^. These characteristics strongly suggest that MAP6 plays a major role in brain anatomy and white matter tract connectivity. The goal of this study was to characterize the changes to brain anatomy in MAP6-KO animals using 3D neuroimaging techniques including Magnetic Resonance Imaging (MRI) and fluorescence microscopy on cleared brains. Images were acquired at high spatial resolution to allow detection and differentiation of neuronal tracts and brain regions in the small mouse brain. The 3D Diffusion Tensor Imaging (DTI) tractography of mouse white matter was validated by confirming results using fluorescence microscopy on cleared brains. The results revealed the basis of the neuroanatomical defects in MAP6-KO mice, in particular in the corticospinal tract, which connects the subset of layer V cortical neurons to motor neurons and interneurons in the spinal cord.

## Results

### MAP6 deletion affects brain anatomy

**Figure 1:**
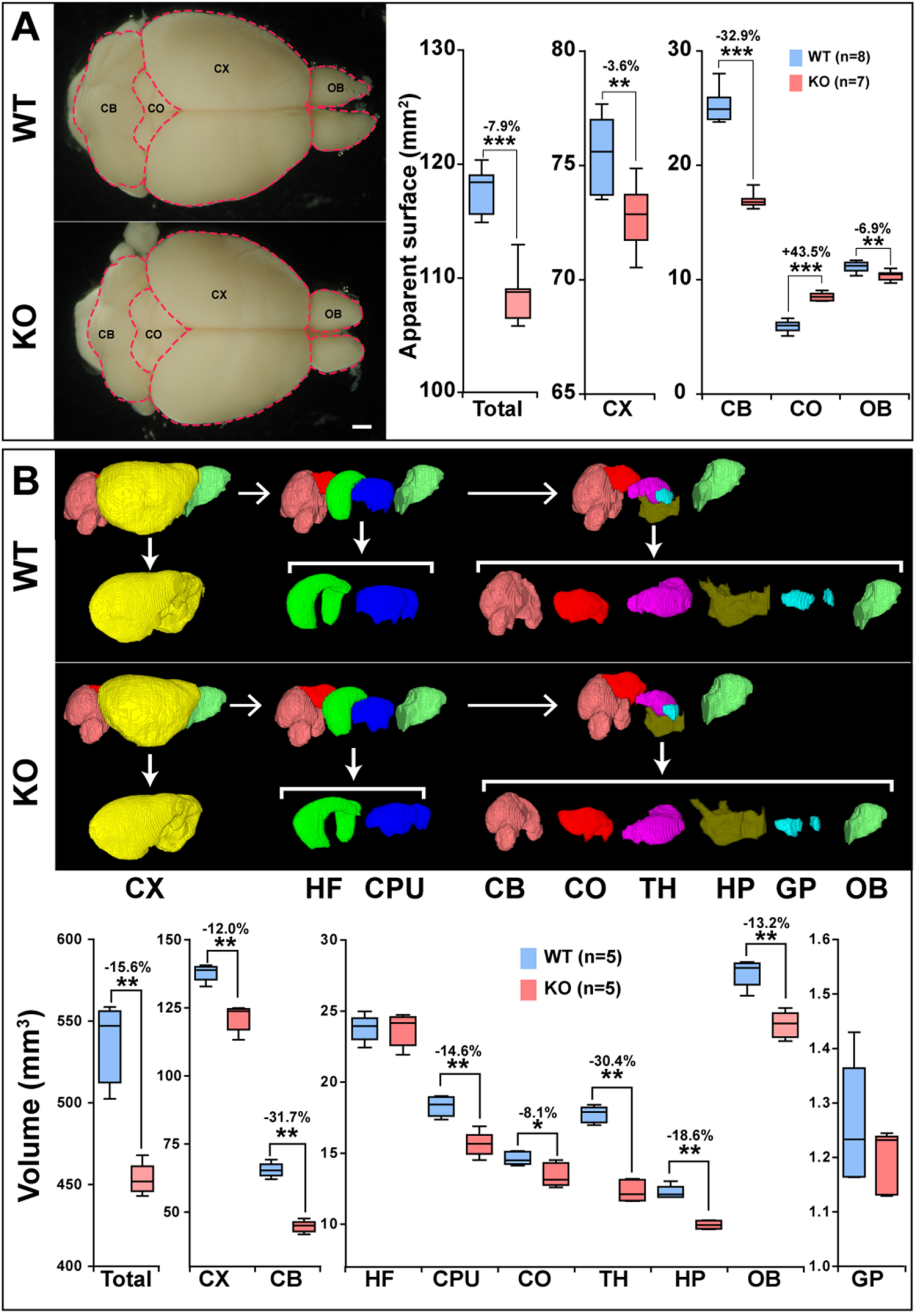
Morphological macroscopic defects associated with MAP6 deletion. (**A**) WT and MAP6-KO brains photographed on dorsal views; apparent brain area was manually delimited (red line). Scale bar: 1 mm. The box-and-whisker plots show the apparent brain area for different regions in mm^2^. Significant percent changes (decrease or increase) are indicated by asterisks. (**B**) 3D reconstructions of brain areas were built from high spatial resolution MRI-T_1w_ data. Each brain structure is represented with a specific color: cortex (CX, yellow); hippocampal formation (HF, dark green); caudate putamen (CPU, dark blue); cerebellum (CB, pink); colliculi (CO, red); thalamus (TH, purple); hypothalamus (HP, khaki); globus pallidus (GP, light blue); olfactory bulbs (OB, light green). The box-whisker plots show volumes for these regions (in mm^3^). Percent volume reduction in MAP6-KO mice is indicated. Black bars represent the median, boxes represent the 25^th^ and 75^th^ percentiles, and the whiskers represent the 5^th^ and 95^th^ percentiles (**p < 0.01; ***p < 0.001, Mann-Whitney test).

All the results given here are discussed as comparisons between results from MAP6-KO and wild type (WT) mice. Figure 1A shows a macroscopic analysis of the apparent brain surface. Surface quantifications for brain structures in WT and MAP6-KO were compared on dorsal macroscopic views. Significant disparities were observed between brain surface areas for MAP6-KO to WT. Thus, in MAP6-KO animals, the apparent surface of the whole brain was decreased (−7.9 ± 1.9%, *p* = 0.0040), as were those of the cortex (CX, −3.6 ± 1.8%, *p* = 0.0093), the olfactory bulbs (OB, −6.9 ± 3.8%, *p* = 0.0059) and the cerebellum (CB, −32.9 ± 2.6%, *p* = 0.0040). In contrast, the surface areas of the superior and inferior colliculi regions were increased (CO, + 43.5 ± 6.0%, *p* = 0.0040).

Figure 1B shows brain structure segmentation and their corresponding volume quantifications, as determined from 3D T_1W_ MRI data. The overall brain volume was decreased by 15.6 ± 1.7% (Total, *p* = 0.0079) but the decrease was not uniform. Thus, a marked decrease in volume was noted for the cerebellum (CB, −31.7 ± 3.2%, *p* = 0.0079) and the thalamus (TH, −30.4 ± 4.2%, *p* = 0.0079), whereas the reduction was less substantial for the cortex (CX, −12.0 ± 3.2%, *p* = 0.0079), the striatum (CPU, −14.6 ± 4.7%, *p* = 0.0079), the hypothalamus (HP, −18.6 ± 2.3%, *p* = 0.0079) and the olfactory bulbs (OB, −13.2 ± 3.3%, *p* = 0.0079). The volume of the colliculi (CO, −8.1 ± 5.6%, *p* = 0.0556), the globus pallidus (GP, *p* = 0.3095) and the hippocampal formation (HF, *p* = 1.0000) were unchanged by lack of MAP6. Altogether, these results show a clear reduction in total brain volume in MAP6-KO mice, which is associated with heterogeneous anatomical changes. A drastically reduced volume was measured in the cerebellar and thalamic regions, while hippocampal formation was unaltered.

### 3D DTI and fluorescence microscopy on cleared brains

**Figure 2:**
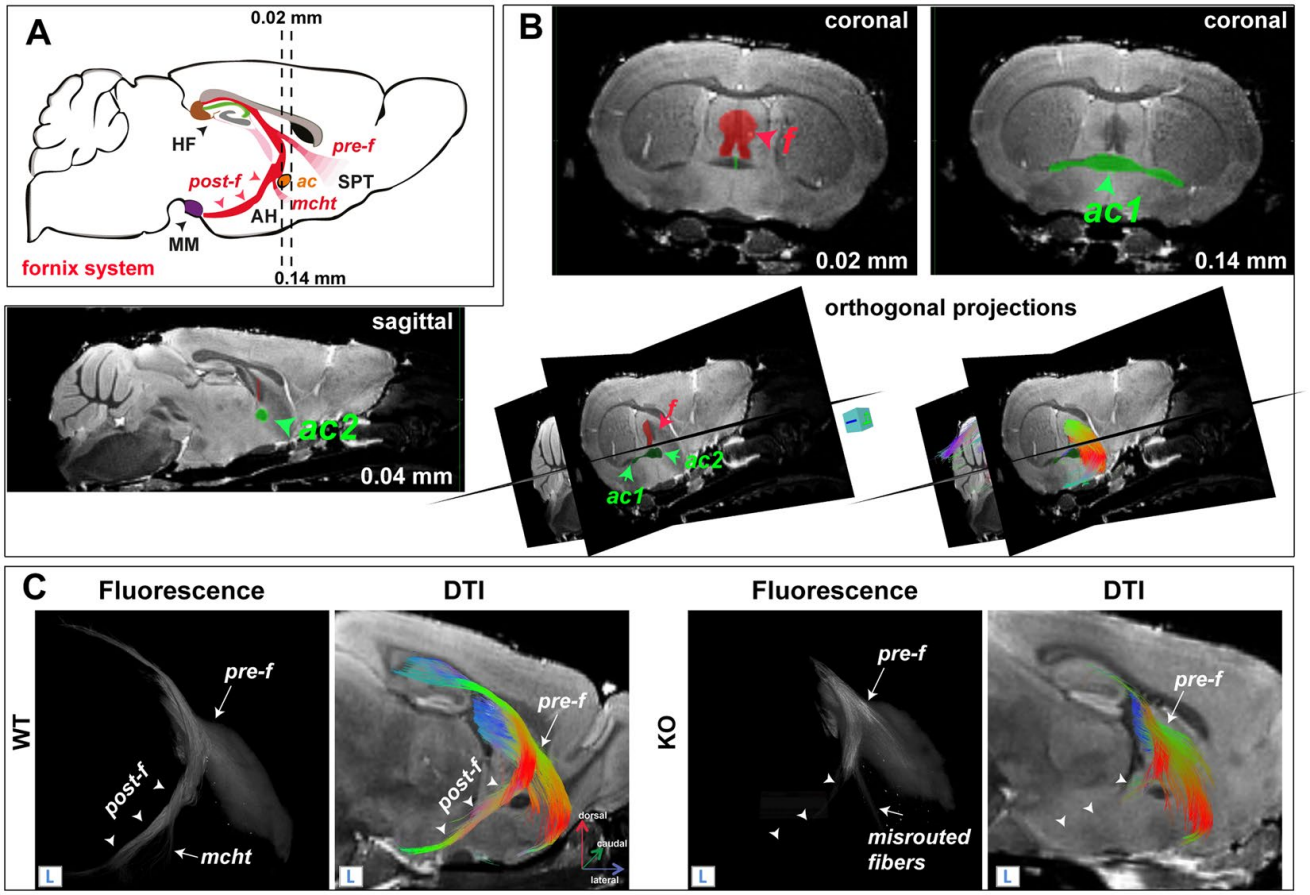
Fornix tract reconstruction from DTI- and fluorescence microscopy on cleared brains. (**A**) Schematic representation of the fornix tract (red region) on a sagittal diagram. Dashed lines indicate the Bregma position, in mm of the coronal planes. HF: hippocampal formation; MM: mammillary body; AH: anterior hypothalamus; SPT: septum; pre-f: pre-commissural fornix; post-f: post-commissural fornix; mcht, medial-cortico-hypothalamic tract and ac: anterior commissure. (**B**) The tractography of the fornix tract is shown on reference diffusion images. The tract was reconstructed using a restricting ROI (coronal, red, f) over the fimbria-fornix and two excluding ROIs (green) drawn on the coronal (coronal, green, ac1) and mid-sagittal (sagittal, green, ac2) planes, over the anterior commissure. (**C**) 3D-DTI tractography of the fornix compared to data acquired by fluorescence microscopy on cleared brain. The two imaging modalities show similar results and detect the absence of the post-commissural fornix in MAP6-KO mice (arrowheads). These results validate our 3D DTI method for WM tractography.

Figure 2 shows the fornix tract reconstructed from DTI data compared to the tract defined by fluorescence microscopy on cleared brains. Figure 2A presents the fornix system, which is a major output of the hippocampal formation, schematized in normal brain. This complex neuronal tract is composed of three major efferent tracts: the pre-commissural fornix (pre-f) which is rostral to the anterior commissure (ac) and projects into septal regions (SPT); and the post-commissural fornix (post-f) which passes behind the anterior commissure, innervates the anterior hypothalamus (AH) through the medial-corticohypothalamic tract (mcht) and terminates at the posterior hypothalamus in the mammillary body (MB)^22–26^.

To reconstruct the fornix tract from DTI data, a restricting ROI was drawn around the fimbria-fornix (Fig. 2B, coronal plane, red ROI, Bregma: 0.02 mm; also see supplemental Table 1). Two excluding ROIs were also used to avoid contamination of the trace with nerve fibers from the anterior commissure. These ROIs were placed on the anterior commissure, on the mid-sagittal plane (Fig. 2B, sagittal plane, green ROI, lateral −00.4 mm) and on the coronal plane (Fig. 2B, coronal plane, green ROI, Bregma: 0.14 mm).

**Table 1:** Perpendicular ADC (ADC_⊥_) and geometric parameters for some key tracts (n = 8 WT and n = 8 KO mice; *p < 0.05, **p < 0.01, ***p < 0.001; Mann-Whitney test).

neuronal tracts	Volume (mm^3^)		Number of fibers		Mean length (mm)		Mean ADC⊥	
	WT	KO	WT	KO	WT	KO	WT	KO
anterior commissure (ac)	3.3 ± 0.5	1.5 ± 0.2***	1285 ± 207	673 ± 90***	5.6 ± 0.5	4.6 ± 0.4**	0.44 ± 0.03	0.41 ± 0.04
intrabulbar anterior commissure (aci)	3.8 ± 0.3	2.3 ± 0.6***	1420 ± 200	1157 ± 432**	3.3 ± 0.4	2.6 ± 0.3***	0.49 ± 0.03	0.47 ± 0.05
corpus callosum (cc)	27.4 ± 1.0	15.5 ± 1.5***	15423 ± 1355	7561 ± 1099***	6.6 ± 0.8	5.4 ± 0.3*	0.42 ± 0.02	0.42 ± 0.03
cerebral peduncle (cp)	16.1 ± 1.7	11.1 ± 0.9***	7693 ± 656	5642 ± 493***	4.2 ± 0.3	3.4 ± 0.1***	0.43 ± 0.02	0.42 ± 0.02
fornix system (f)	15.4 ± 12.0	10.5 ± 1.3***	5120 ± 982	3646 ± 857*	6.7 ± 0.5	6.0 ± 0.4*	0.40 ± 0.02	0.40 ± 0.03
fasciculus retroflexus (fr)	0.98 ± 0.19	0.79 ± 0.23	393 ± 58	293 ± 46**	2.3 ± 0.1	2.1 ± 0.3	0.44 ± 0.02	0.43 ± 0.03
internal capsule (ic)	20,8 ± 1.9	12.7 ± 1.9***	9997 ± 1546	5991 ± 1182***	3.8 ± 0.3	3.1 ± 0.1***	0.43 ± 0.02	0.42 ± 0.03
mammillary tract (mt)	0.91 ± 0.20	0.46 ± 0.2***	243 ± 54	123 ± 35***	1.9 ± 0.2	1.7 ± 0.3	0.45 ± 0.01	0.44 ± 0.04
optic tract (opt)	6.9 ± 1	3.9 ± 0.7***	2453 ± 407	1315 ± 301***	5.4 ± 0.5	4.4 ± 0.3**	0.49 ± 0.02	0.45 ± 0.03
pyramidal tract (py)	6.1 ± 1.6	3.0 ± 0.8***	1546 ± 473	1014 ± 304*	3.8 ± 0.5	3.1 ± 0.4*	0.44 ± 0.01	0.39 ± 0.09
stria medularis (sm)	1.53 ± 0.32	0.72 ± 0.09***	554 ± 61	330 ± 53***	2.6 ± 0.3	1.9 ± 0.19***	0.40 ± 0.03	0.41 ± 0.04
stria terminalis (st)	2.7 ± 0.5	2.0 ± 0.4**	682 ± 162	508 ± 126*	3.7 ± 0.7	3.3 ± 0.5	0.40 ± 0.02	0.39 ± 0.03

The comparison of reconstructions of the fornix system derived from DTI tractography and fluorescence microscopy data on cleared brains (Fig. 2C) show that tract reconstructions in WT and MAP6-KO mice were similar with the two techniques excepted for the medial-corticohypothalamic tract (mcht), which projects toward the anterior hypothalamus, and misrouted fibers cannot be visualized by DTI tractography (Fig. 2C, Movie 1).

### Heterogeneous alterations to neuronal tracts

**Figure 3:**
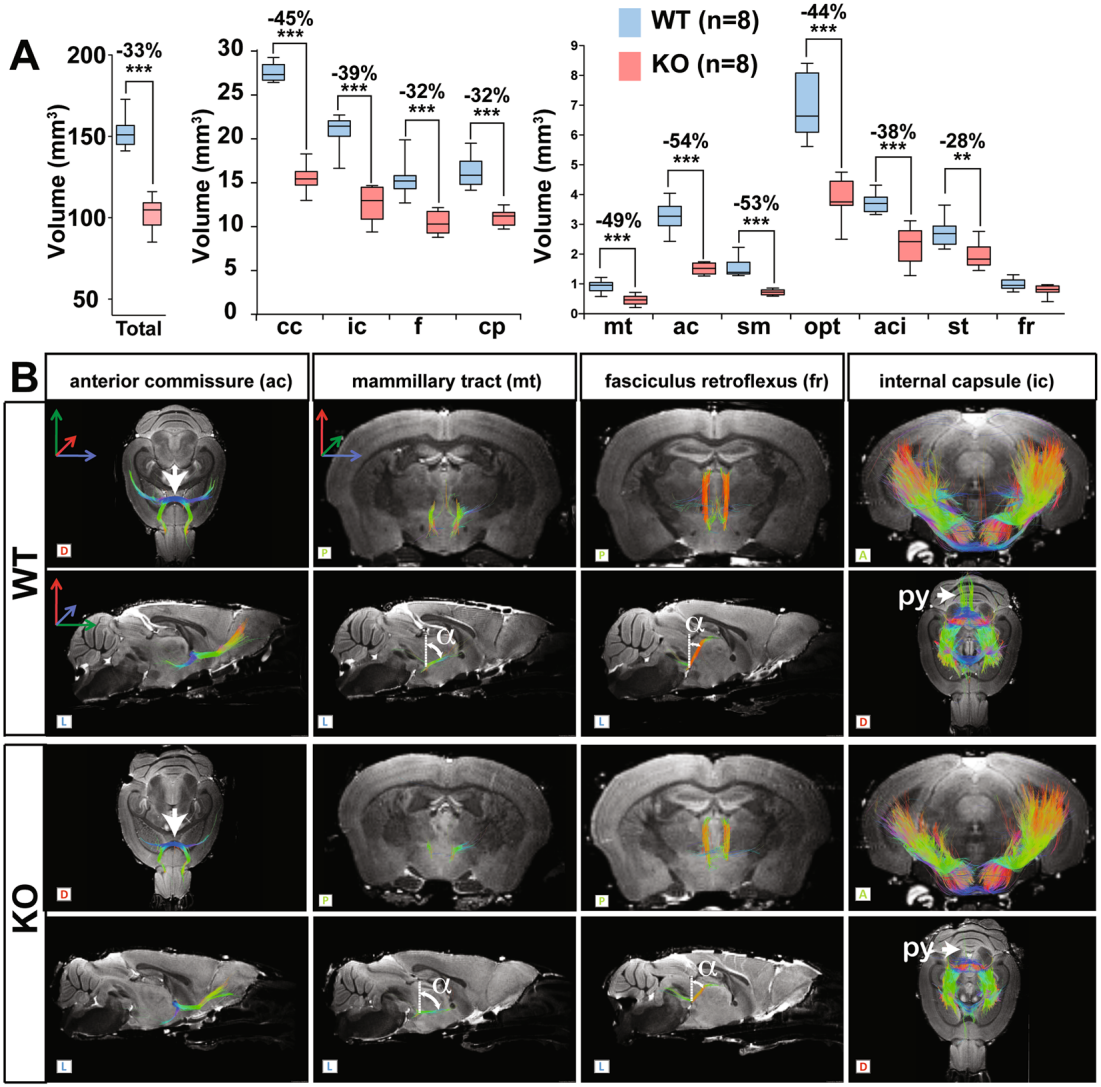
DTI tractography shows heterogeneous alterations neuronal tracts in MAP6-KO mice. (**A**) Neuronal tract volumes. Volume reductions in MAP6-KO mice are indicated in percent when significant. Black bars represent the median, boxes represent the 25^th^ and 75^th^ percentiles, and whiskers represent the 5^th^ and 95^th^ percentiles (**p < 0.01; ***p < 0.001, Mann-Whitney test). (**B**) 3D DTI tractography reconstructions of reference diffusion images. The letters L (lateral), D (dorsal), V (ventral), P (posterior) and A (anterior) indicate the viewing position on the display screen. The anterior commissure (ac), the mammillary tract (mt), the fasciculus retroflexus (fr) and the internal capsule (ic) are indicated. The ac was thinner in MAP6-KO brains than in WT brains (arrows). The mt and the fr exhibit structural modifications with a modified inclination angle (α). In MAP6-KO mice, the pyramidal projections were dramatically reduced (arrow, py).

**Figure 4:**
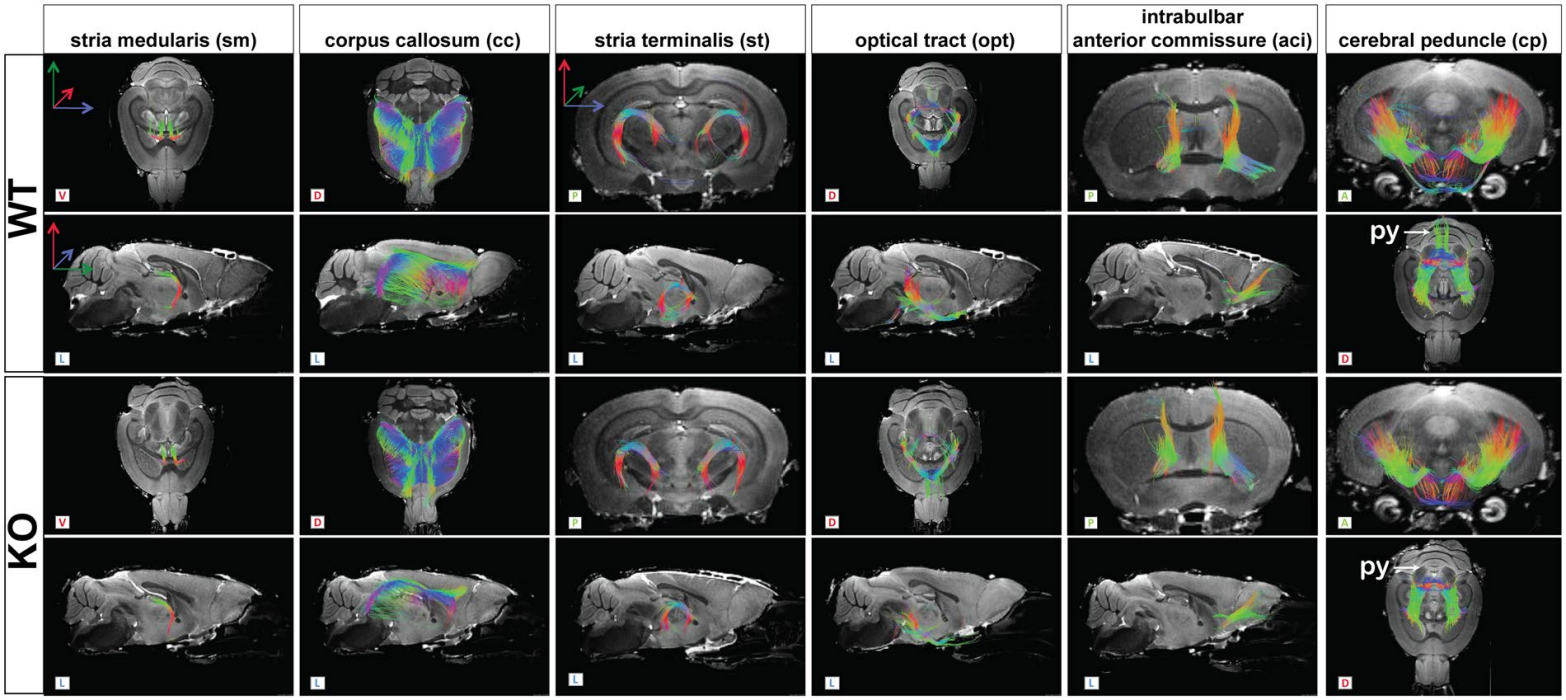
3D DTI tractography reconstructions of several key neuronal tracts. The letters L (lateral), D (dorsal), V (ventral), P (posterior) and A (anterior) indicate the viewing position on the display screen. In the tractography for the cerebral peduncle, the white arrows indicate the pyramidal projections (py).

Figures 3 and 4 summarize the tract sizes determined for various brain structures. The volume of white matter, as visualized in whole-brain DTI tractography, was reduced by 33 ± 6.5% (*p* = 0.0002) (Fig. 3A); a similar tendency was observed for the internal capsule (ic, −39 ± 9%, *p* = 0.0002), the fornix (f, −32 ± 8%, *p* = 0.0002), the intrabulbar anterior commissure (aci, −38 ± 16%, *p* = 0.0009) and the cerebral peduncle (cp, −32% ± 6%, *p* = 0.0002) (Fig. 3A). The reduction was pronounced for the optical tract (opt, −44 ± 10%, *p* = 0.0009), the mammillary tract (mt, −49 ± 18% *p* = 0.0006), the anterior commissures (ac, −54 ± 5%, *p* = 0.0002), the stria medularis (sm, −53 ± 6% *p* = 0.0002) and the corpus callosum (cc, −44 ± 5% *p* = 0.0002). The size of the fasciculus retroflexus and the stria terminalis was also reduced, but the difference was not significant for the fasciculus retroflexus (fr, −19 ± 18%, *p* = 0.0650 and st, −28 ± 15%, *p* = 0.0047). Interestingly within the anterior commissure, we observed differences between the anterior part of the anterior commissure (aca, −39 ± 10%, *p* = 0.0009) and the posterior part of the anterior commissure (acp, −70 ± 11%, *p* = 0.0009).

For several key tracts we report the volume, their fiber number and length (Table 1). For all these tracts, the volume was reduced mainly because of a reduced number of fibers, but also due to shorter tracts. These two geometrical alterations (number and length of fibers) caused the connections between brains areas to form incompletely, an effect which might underline the severe behavioral disorders observed in MAP6-KO mice. Furthermore, in regions where fibers remained, the mean ADC _perpendicular_ value, which gives an indication of the water diffusion anisotropy, remained unchanged. This result indicates that, even when several key tracts are drastically damaged, the integrity of myelin fiber is not affected.

Representative reconstructions of each neuronal tract illustrating specific alterations are shown in Figs 3B and 4. In these images, the anterior commissure appeared much thinner (Fig. 3B, ac, arrow) and the rectilinear portion of the fasciculus retroflexus was shorter (−24 ± 6%, *p* = 0.0002) and its dorso-ventral position was shifted, as indicated by a significant change in angle of +12 ± 5° (Fig. 3B, fr, α, *p* = 0.0002). In the figures, these alterations are reflected by color changes from red to orange (Fig. 3B, fr). Similarly, deviation of the angle of the mammillary tract increased significantly (Fig. 3B, mt, α, + 15 ± 4°, *p* = 0.0011) without any change to its length (*p* = 0.9163). For these specific tracts, changes in spatial position were clearly visible. Finally, severely decreased posterior projections of the internal capsule (Fig. 3B, ic, white arrow) and of the cerebral peduncle (Fig. 4, cp, white arrow) were detected. These projections are part of the pyramidal tract, which itself contributes to the corticospinal tract.

### Alterations to the corticospinal tract

**Figure 5:**
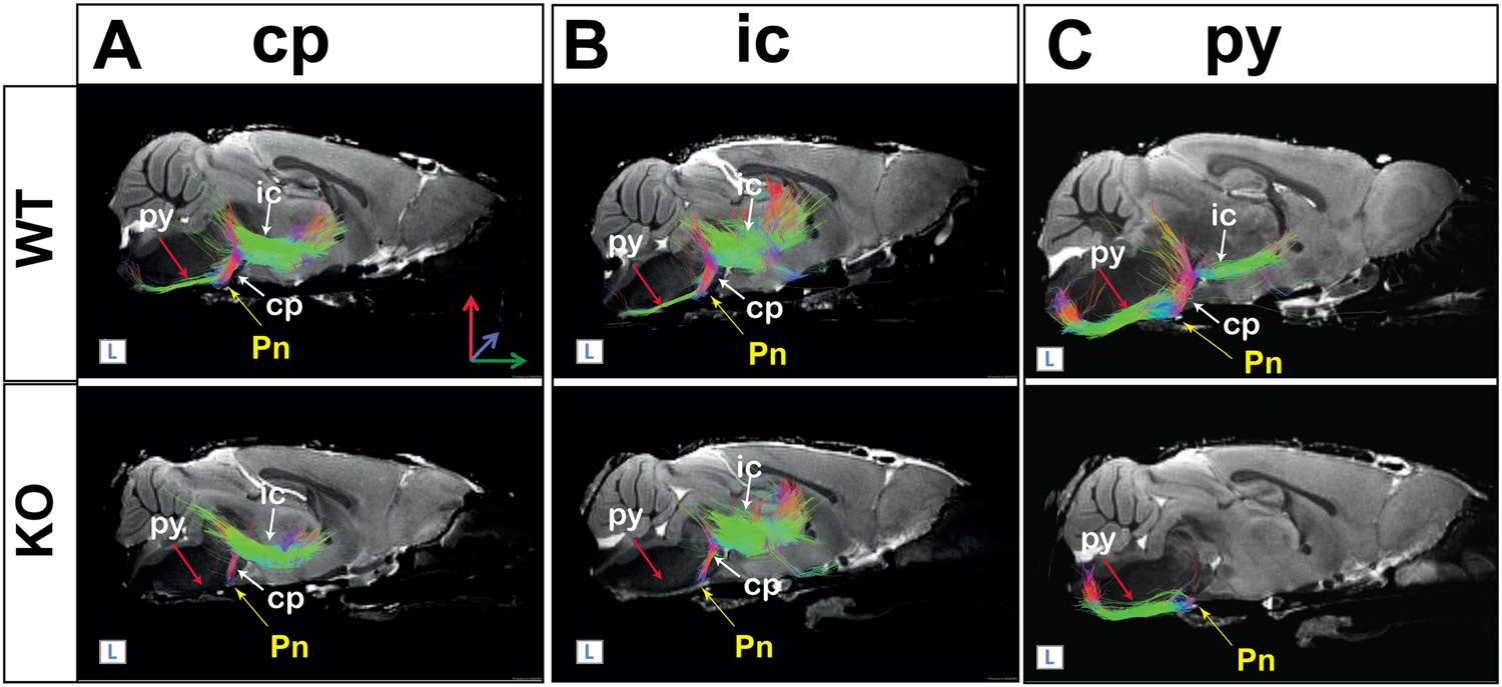
3D DTI tractography reveals disruption of the corticospinal tract. The corticospinal tract is displayed on reference sagittal diffusion images. Corticospinal projections were obtained by applying a restricting ROI within the internal capsule (**A**, ic) and within the cerebral peduncle (**B**, cp) or within the pyramidal tract (**C**, py). In MAP6 KO mice, all reconstructions showed a break-point on the corticospinal tract at level of the pontine nuclei (Pn, yellow arrow).

**Figure 6:**
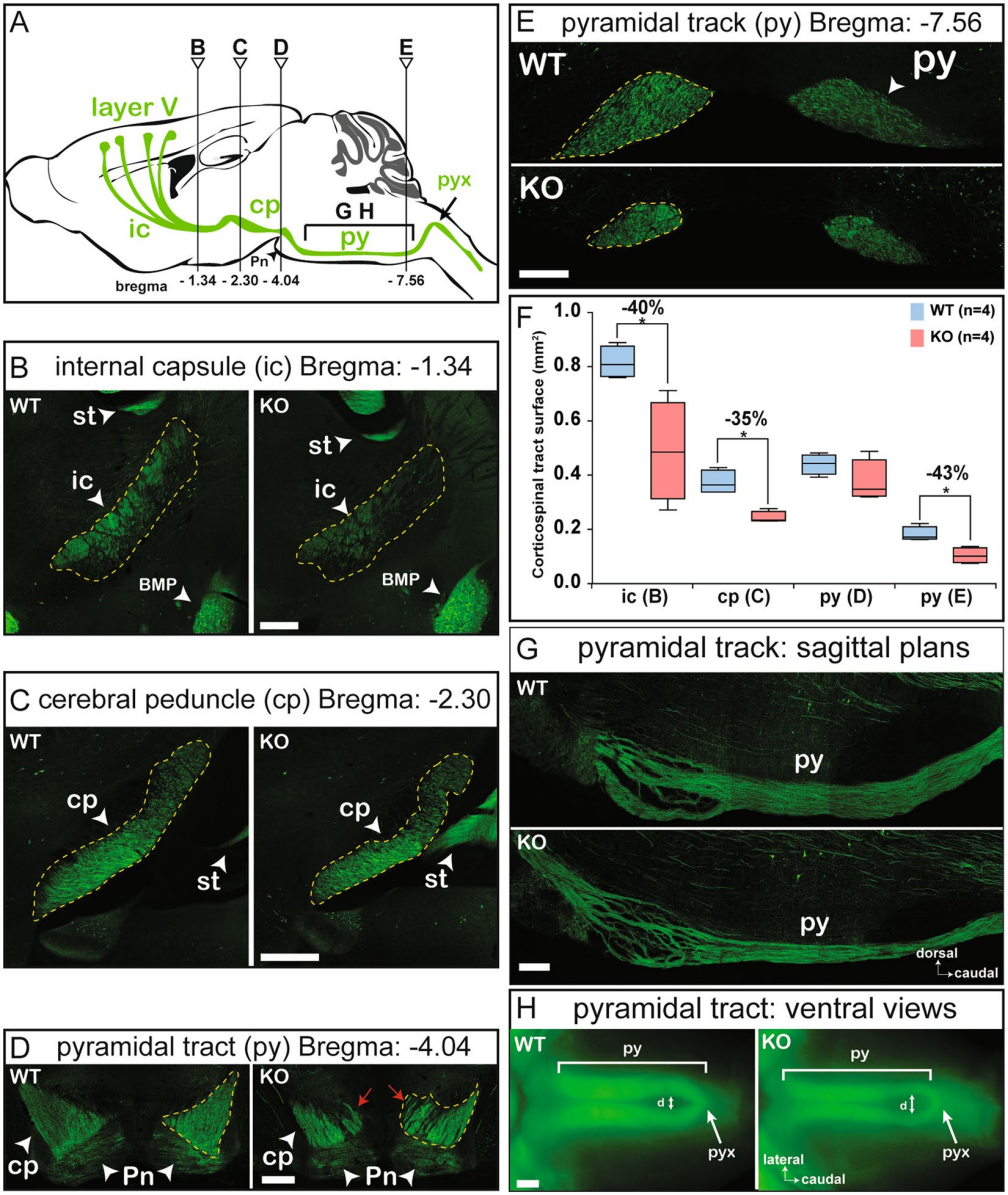
MAP6 deletion affects pyramidal tract integrity. (**A**) Schematic representation of the corticospinal pathway (green) on a sagittal diagram. The corticospinal tract is mostly formed from axonal projections of cortical layer V. These axons successively pass through the internal capsule (ic), the cerebral peduncle (cp) and the pyramidal tract (py) and decussation (pyx), before terminating in the spinal cord. The Bregma in mm and positions for coronal (**B**–**E**) and sagittal planes (**G**), and ventral view (**H**) are indicated. (**B-E**) Coronal images at Bregma −1.34 mm, −2.54 mm, −4.04 mm, and −7.56 mm. In D, red arrows indicate de-fasciculation of the pyramidal tract at the level of the pontine nuclei (Pn). ROIs (dashed yellow lines) delimit the tract area. (**F**) Corticospinal tract area measured at coronal level (seen in **B**–**E**) corresponding to the internal capsule (ic), the cerebral peduncle (cp), the anterior pyramidal tract (py) and the posterior pyramidal tract (py). Black bars represent the median, boxes represent the 25^th^ and 75^th^ percentiles, and the whiskers represent the 5^th^ and 95^th^ percentiles (*p < 0.05; Mann-Whitney test). Other abbreviations used: st, stria terminalis and BMP, amygdala. (**G**) Sagittal sections of the pyramidal tract within the brainstem. The pyramidal tract was much thinner in the posterior region of MAP6-KO mice than in WT brains. (**H**) Ventral views of whole-mount brainstem. Double arrowheads (d) indicate where the interstitial space of the posterior pyramidal tract was measured. Scale bar: 400 µm.

In WT, and as normally described, the corticospinal tract could be accurately visualized by DTI (Fig. 5A,B, cp and ic respectively). The reconstructed fibers projected through the internal capsule (ic) and the cerebral peduncle (cp) toward the brainstem via the pyramidal tract (py) to terminate in the spinal cord. At the boundary between the medulla and the spinal cord, the pyramidal tract turns in the dorsal direction and crosses the midline to form the pyramidal decussation (pyx) (see also diagram in Fig. 6A).

In WT mice, the pyramidal projections were visible (Fig. 5A and Fig. 5B, red arrow). Different restricting ROIs were used, one from the cerebral peduncle (Fig. 5A) and the second from the internal capsule (Fig. 5B). In MAP6-KO brains, the pyramidal projection was clearly lost, as evidenced by a breaking point at the level of the structure containing the pontine nuclei (Fig. 5A,B, Pn, yellow arrow).

Similarly, in MAP6-KO, when reconstructing the corticospinal tract from the pyramidal tract (Fig. 5C, py), we also detected a breaking point at the pontine nucleus (Fig. 5C, Pn, yellow arrow). Taken together, information provided by tractography reconstructions indicated that several corticospinal projections are disrupted at the level of the pontine nucleus in MAP6-KO mice.

The full corticospinal tract was visualized in Thy-eYFP-H mice by fluorescence microscopy (Fig. 6). This visualization was possible since this tract is mostly composed of axonal projections of cortical neurons from layer V that specifically express eYFP in this model (Fig. 6A)^27^. Coronal sections were used to compare the general structure of the corticospinal tract between WT and MAP6-KO mice. We initially observed that the area of the internal capsule (Fig. 6B) and of the cerebral peduncle (Fig. 6C) were decreased by 40 ± 22% and 35 ± 6%, respectively (Fig. 6F, ic, *p* = 0.0286 and cp, *p* = 0.0286); subsequently, we noted a severe defasciculation of the anterior part of the pyramidal tract at the level of the pontine nuclei (Fig. 6D, red arrows). In WT mice, the pyramidal tract forms a compact triangle; which is not the case in MAP6-KO (see red arrows, Fig. 6D). We further examined the posterior part of the pyramidal tract that was reduced by 43 ± 16% (Fig. 6E,F, *p* = 0.0286). The examination of the general appearance of the pyramidal tract on whole-mount brainstem (Fig. 6G) and sagittal sections (Fig. 6H) confirmed this atrophy. In MAP6-KO mice, the pyramidal tract was significantly thinner and the interstitial space of the posterior pyramidal tract was much broader than in WT animals (+67%, *p* = 0.0011) (Fig. 6G, d).

Altogether, data from DTI tractography and fluorescence microscopy imaging indicated a severe restriction of the corticospinal tract with evidence of defasciculation at the location of the pontine nuclei.

## Discussion

In this article we used 3D-MRI imaging techniques to characterize the anatomical defects present in MAP6-KO mouse brains. Our results show that 3D DTI tractography can contribute to our understanding and assist with investigations into the structural connectivity of the neuronal tracts in rodents, primates and humans for basic, translational and clinical research purposes^28, 29^. In mouse models, the very small brain volume is a real limitation to achieving high spatial resolution in a reasonable time-frame as the signal-to-noise ratio achievable for a single image voxel tends to be low. For the purposes of this study, DTI was performed *ex vivo* using exogenous gadolinium-based MRI contrast agents^30, 31^ to reduce the relaxation times, in particular the longitudinal value T_1_. In these conditions, acquisition time was accelerated, and a high isotropic spatial resolution (80 µm) was attained with a signal-to-noise ratio of 104. For this imaging, diffusion gradients were used in only six spatial directions. This scheme was preferred to HARDI (High Angular Resolution Diffusion Imaging) acquisition, which uses higher numbers of spatial gradient diffusion directions, since in preliminary studies comparing the two methods white matter reconstructions were similar with the same total acquisition time (data not shown). To note separate cohorts of mice were used for volumetric and DTI acquisitions due to the design of the protocol and the availability of magnets; each set of experiments giving significant differences between WT and MAP6-KO animals.

One of the major results of our study is the validation of the 3D DTI tractography by the fluorescence microscopy on cleared brains. Comparison of reconstructions of the fornix system derived from DTI tractography and fluorescence microscopy data on cleared brains (Fig. 2C) showed that tract reconstructions in WT and MAP6-KO mice were similar with the two techniques excepted for the medial-corticohypothalamic tract (mcht). This may be related to the lower spatial resolution of DTI compared to optical microscopy (80 µm versus 1.66 µm).

One of the major findings of this study related to the general appearance of the whole brain observed by macroscopic analysis of the apparent surface area in MAP6-KO mice and the marked reductions in volume of the thalamus, the cerebellum and the cerebral cortex. In contrast, the hippocampus was not affected. These results are consistent despite the relatively small number of animals analyzed, since the SD and *p* values were small, indicating a high penetrance of phenotypic changes in MAP6-KO mice. The severely decreased volumes of the cerebellum, the thalamus and the cortex probably cause the new brain organization observed MAP6-KO mice. This new organization affects the apparent surfaces of the colliculi (Fig. 1A) as well as the spatial position of the mammillary and retroflexus tracts (Fig. 3B). Regarding the colliculi, in MAP6-KO mice, we observe a major difference between the small reduction of their volumes and the strong enlargement of their apparent surfaces. This difference results from the combined reduction of cerebellum and cortex uncovering the colliculi and thereby changing their external surface appearance. Overall there was no obvious changes in colliculi shape (Fig. 1B). Altogether, these results indicate that the overall reduction in brain volume is not uniform, and that it affects specific brain structures to different extents. A recent study comparing wild and domestic chickens showed that changes to the volumes of brain regions evolve independently^32^, in accordance with the “mosaic evolution” hypothesis^33^. Indeed, Barton *et al*. identified several loci responsible for the heterogeneous variations in mass reported during the chicken domestication, and MAP6 was shown to be related to the locus controlling cerebellum mass; an observation which is in line with our findings.

It is quite surprising that the hippocampal formation volume is not affected in MAP6 null animal (this study and ref. 14) despite a strong expression of MAP6 in developing and adult hypoccampus^34^. It might be due to some compensation events carried out by MAP6 related proteins such as MAP6-D1 (SL21) known to be expressed during late brain development^35^.

The anatomical changes noticed in MAP6-KO mice may be linked to the behavioral and cognitive impairments previously reported^9–17, 19, 20^. Indeed, defects in the circuitry connecting the thalamus - which plays a key role in information processing - to the cortex and the cerebellum could explain a number of the schizophrenia-like characteristics of MAP6-KO mice^36–39^. In addition, the cerebellum was initially considered to play a role only in planning and executing movements; however, our results corroborate some recent studies indicating that the cerebellum is also implicated in many psychiatric disorders such as attention deficit hyperactivity disorder, autism spectrum disorders, schizophrenia, bipolar disorder, major depressive disorder, and anxiety disorders^36, 40, 41^.

For the white matter (WM), an overall reduction in volume of 33% was found, in accordance with our pervious findings^21^. However, the loss of white matter was not homogeneously distributed among neuronal tracts, and some tracts were more affected, such as the anterior commissure, the corpus callosum, the mammillary tract and the stria medularis. Regarding the anterior commissure, our results showed that the posterior part is more altered than the anterior part. This result is in agreement with a recent study showing greater defects of the posterior part of the anterior commissure in the Gclm-KO mice, a model of psychiatric disorders^42, 43^. Some tracts (the post-commissural fornix and the corticospinal tract) were severely affected, a result which could explain why the fractional parameter (FA) of white matter was not found to be uniform among all neuronal tracts in MAP6-KO brains^21^. However, the perpendicular ADC (ADC_⊥_) was found to be unchanged (Table 1) suggesting that the myelin fiber sheets are unaffected, a result which is in agreement with those obtained by electron microscopy in our previous work^21^.

DTI tractography further showed that the axonal projections of the pyramidal tract were disrupted at the level of the pontine nuclei. To further investigate this observation, we performed a histological study of the pyramidal tract which revealed a severe defasciculation at pontine level (Fig. 6D). The corticospinal tract is the major descending pyramidal tract connecting a subset of layer V cortical neurons to motor neurons and interneurons in the spinal cord. This tract controls voluntary body movement and fine motor functions of the limbs. MAP6-KO mice have been reported to exhibit motor coordination problems associated with poor performance on the rotarod task^10^. This phenotype could be explained by disruption of corticospinal tract. Interestingly; many MRI studies reported alterations of white matter structures belonging to the corticospinal tract in schizophrenic patients^44–47^. Bilateral decrease of FA in pyramidal and corticopontine tracts was strongly associated with motor performance deficits in adolescent-onset schizophrenia^47^ suggesting that functional deficits in schizophrenia are not only confined to social and cognitive domains^48^.

## Conclusion

In this study, 3D DTI tractography was useful to detect major defects in brain connections in MAP6 KO mice. The results highlighted several differences in neuroanatomical volumes and apparent surfaces, as well as abnormalities in tractography present in several key brain regions. The study especially highlights a severe damage of the corticospinal tract with defasciculation at the location of the pontine nuclei. The major neuroanatomical defects obtained here could be considered as psychiatric biomarkers and should help in clinical environment to establish possible correlations between the neuroanatomy changes and specific psychiatric disorders to establish links between brain disconnection and impairments of integrated brain function.

## Materials and Methods

### Ethical approval and mice

All experiments involving animals were conducted in accordance with the policy of the Institut des Neurosciences of Grenoble (GIN) and the French legislation; experiments were done in compliance with the European Community Council Directive of 24 November 1986 (86/609/EEC). The research involving animals was authorized by the Direction Départementale de la protection des populations—Préfecture de l’Isère-France (Deloulme J. C., PhD, permit number 380822) and by the ethics committee of GIN n° 004 accredited by the French Ministry for of Research. MRI experiments were performed on animals with a 129SvPas/C57BL6-F1genetic background while fluorescence microscopy on brain slices and on cleared brains were performed on animals with a 129SvPas/C57Bl6-Thy1-eYFP-H-F1 genetic background. The homogeneous inbred 129SvPas/C57BL6-F1 WT and MAP6 KO mice were generated by crossing pure 129SvPas MAP6-heterozygous mice with pure C57BL6 MAP6-heterozygous mice. The homogeneous inbred 129SvPas/C57Bl6-Thy1-eYFP-H-F1 mice were generated by crossing pure 129SvPas MAP6-heterozygous mice with pure C57BL6-Thy1-eYFP-H MAP6-heterozygous mice. All experiments were conducted on WT or MAP6-KO adult (3–6 months) female or male littermates. To note, although a 2.5% larger whole brain volumes has been reported in C57Bl6 males as compared to females^49^ in our study with the samples size, no significant difference between sex was found.

For this study, a total of 53 adult mice were used: 31 WT and 30 MAP6-KO. Animals were placed in study groups as follows: 8 WT and 7 MAP6-KO mice for the stereo-macroscopic analysis; 5 WT and 5 MAP6-KO for volumetric MRI; 8 WT and 8 MAP6-KO for DTI tractography; 2 WT and 2 MAP6-KO for fluorescence microscopy on cleared brain; 8 WT (4 sliced in coronal sections and 4 in sagittal sections) and 8 MAP6-KO (4 sliced in coronal sections and 4 in sagittal sections) for corticospinal tract histology.

### Brain preparation for *ex vivo* MRI acquisitions

Brains were fixed before harvesting from animals by transcardiac perfusion of a paraformaldehyde solution 4% in phosphate buffered saline, to which 6.25 mM of Gd-DOTA was added (Guerbet Laboratories) as contrast agent. The contrast agent was used here to accelerate MRI acquisition time. After removing surrounding skin and muscles, the skulls containing intact brains were immersed in the fixing solution for four days, and then transferred to a Fomblin (FenS chemicals) bath for at least eleven days after brain fixation. This schedule provided homogeneous distribution of the Gd-DOTA throughout the whole brain^31^.

### MRI acquisitions

*Ex vivo* 3D MRI acquisitions at high spatial resolution were performed at 7 T and 9.4 T (Bruker Biospec Avance III) for DTI and T_1w_ acquisitions respectively, both using a volume coil for transmission and ahead surface coil for reception.

A 3D T_1W_ gradient-echo MRI sequence was used for brain segmentation and volumetric analysis with a repetition time of 27.5 ms, an echo time of 5.5 ms, and a flip angle of 20°. The field of view was set to 10.8 × 7.44 × 15.24 mm^3^, which produced an isotropic spatial resolution of 60 µm. The number of signal accumulations was 6, and the total acquisition time was 1 h 40 min for each brain.

3D spin-echo DTI sequence with Cartesian K-space sampling was used for WM imaging and analysis. This sequence was preferred to the rapid echo planar imaging (EPI) sequence as it provided a high isotropic spatial resolution of 80 µm while avoiding potential geometric distortions and susceptibility artifacts in a field of view measuring 20.48 × 8.5 × 11.5 mm^3^. The repetition time, echo time and number of accumulations were set to 90 ms, 16 ms and 22 ms, respectively. Diffusion gradients were applied with duration of δ = 3.5 ms and a separation duration Δ = 8 ms, in 6 different spatial orientations [1, 1, 0]; [0, 1, 1]; [1, 0, 1]; [1, −1, 0]; [0, 1, −1]; [−1, 0, 1] with a gradient factor (b-value) of 1500 s/mm^2^. The reference diffusion image was acquired with a low b-value (50 s/mm²). The total duration of the microscopic 3D DTI scan was 59 h.

### Analysis of brain volumes

Brain regions were defined by MRI segmentation on the 3D T_1w_ MR images. Each cerebral structure was manually delimited using regions of interest (ROI) drawn every five slices on the coronal orientation of the 3D T_1w_ MRI, using Fiji software^50^. Using the Segmentation editor plug-in (http://fiji.sc/Segmentation_Editor) the whole cerebral structure could thus be reconstructed by interpolation between ROIs. Then, all slice were manually corrected based on the mouse brain atlas^51^. Measurements were obtained for the following structures: the caudate putamen (CPU), the cerebellum (CB), the colliculi (CO), the cortex (CX), the globus pallidus (GP), the hippocampal formation (HF), the hypothalamus (HP), the olfactory bulb (OB) and the thalamus (TH). Each structure was color-coded, and its 3D representation and volume were determined using the 3D viewer plug-in in the Fiji software (https://imagej.net/3D_Viewer). For each region, the volume was calculated as the number of voxels × the voxel volume.

### DTI processing and fiber tracking

The 3D tractography of WM fibers was computed using the Tensor Toolkit software (https://gforge.inria.fr/projects/ttk) after suppressing background noise by applying the following parameters: initial FA_1_ value = 0.4, and a cutoff FA_2_ value = 0.28. A whole-brain tractogram was then obtained using one seed point per voxel, i.e., 1950 seeds per mm^3^.

3D fiber tractography was performed using the DTI track module provided in MedINRIA software (version 1.9.4; http://med.inria.fr/)^52^. Each fiber tract structure was derived from the whole-brain tractogram by restricting streamlines with waypoint ROIs drawn manually on reference diffusion images, as described in Fig. 2B. Two types of ROIs were defined to generate each streamline; one was used to select the fibers (defined here as the restricting ROI), while the other was used to exclude fibers potentially contaminating the selected tract (defined here as the excluding ROI). Fiber statistics such as tract volume, fiber number, mean length and apparent diffusion coefficients ADC were also determined using MedINRIA software. Twelve tracts were mapped (see Supplementary Table 1) using this reconstruction strategy. Reconstructed tracts are represented with a color-coded spatial direction, where blue corresponds to the medial-lateral, red to the dorso-ventral, and green to the caudo-rostral directions. To assess deviation of the mammillary tract (mt) and the fasciculus retroflexus (fr) tract in MAP6 KO mice, we used the angle between the axis of the tract and the vertical axis of the image on sagittal planes. This angle was calculated using ImageJ software.

### Macroscopic analysis of apparent surface

Fixed brains were photographed using a stereo-microscope (Leica EZ4HD) and the surfaces of cerebral structures, here called apparent surfaces, were manually delineated on photographs using Fiji software^50^.

### Fluorescence microscopy on brain slices and on cleared brains

All the neuroanatomical abbreviations used in this study were taken from Paxinos’s atlas^51^, except for pre-f, pre-commissural fornix; post-f, post-commissural fornix; AH, anterior hypothalamus; SPT, septum; HF, hippocampal formation; CX, cortex; CB, cerebellum; MB, mammillary bodies; OB, olfactory bulb; TH, thalamus, HP, hypothalamus; CO, colliculi.

#### *Clearing* method

To analyze the fornix system, fixed brains from WT and MAP6-KO C57BL6/129SvPas/Thy1-eYFP mice were bisected at the mid-sagittal plane. Both middle-hemispheres were then cleared using the CUBIC method^53^. Each middle-hemisphere was incubated in Cubic1 reagent (25% urea, 25% N,N,N’,N’-tetrakis (2-hydroxypropyl) ethylenediamine and 15% Triton X-100) for 10 days at 37 °C under gentle agitation. The full half-fornix was imaged on a confocal microscope (Zeiss, LSM 710) using a 10x objective with a voxel resolution of x = 1.66 µm; y = 1.66 µm; and z = 6.5 µm (Grenoble Photonic Microscopy Facility PIC-GIN). The full half-fornix was then reconstructed using the Fiji 3D stitching plug-in (http://fiji.sc/Stitching_2D/3D).

#### Fluorescence microscopy of the corticospinal tract

Fixed 129SvPas/C57Bl6-Thy1-eYFP brains were cut into 40-µm slices using a cryo-microtome (Microm, H560S); slices were mounted on Superfrost plus slides (Menzel-Gläser). Surface quantifications were performed on coronal slices. Epifluorescence of sections was digitized using an Axioscop microscope (Zeiss) equipped with a 5x dry objective and an EMCCD Quantum camera controlled by Metamorph software (Roper Scientific). ROIs were manually drawn and surface areas were calculated using ImageJ software. Since the cerebral volume for WT and KO mice was not the same^21^, the area of the corticospinal tract was quantified on coronal brain sections selected based on morphometric landmarks. Thus, slice B corresponded to the end of the dentate gyrus (Bregma: −1.34 mm); slice C corresponded to the start of the habenular commissure (Bregma: −2.30mm); Slice D corresponded to the end of cerebral peduncle lateralization and the start of the rubrospinal tract (Bregma: −4.04 mm); and slice E corresponded to the 10^th^ slice before the appearance of pyramidal tract decussation (Bregma: −7.56 mm). Epifluorescence for the pyramidal tract was further analyzed on sagittal sections using a confocal microscope (LSM 710, Zeiss), or in whole-mount brainstems using a stereo-microscope (Olympus SZX12).

### Measures and Statistical analyses

All analyses involving manual processing procedures have been performed by at least two persons who were blinded to genotype information. Statistical analyses were performed using Graphpad Prism (V 5.02). A Mann-Whitney test (Two tailed, CI of 95%) was used to compare data series. Values were considered significant when *p < 0.05, **p < 0.01 and ***p < 0.001. All results are given as mean ± SD (standard deviation). All box-and-whisker plots show 5–95% percentiles.

## Electronic supplementary material

Supplementary Information

Supplementary Movie 1

## References

[1] Tischfield MA, Cederquist GY, Gupta ML, Engle EC (2011). Phenotypic spectrum of the tubulin-related disorders and functional implications of disease-causing mutations. Curr. Opin. Genet. Dev..

[2] Bradshaw, N. J. & Hayashi, M. A. F. NDE1 and NDEL1 from genes to (mal)functions: parallel but distinct roles impacting on neurodevelopmental disorders and psychiatric illness. *Cell. Mol. Life Sci*. **74**, 1191–1210 (2017). 10.1007/s00018-016-2395-7 PMC11107680 27742926

[3] Marchisella F, Coffey ET, Hollos P (2016). Microtubule and microtubule associated protein anomalies in psychiatric disease. Cytoskeleton.

[4] Bradshaw NJ, Porteous DJ (2012). DISC1-binding proteins in neural development, signalling and schizophrenia. Neuropharmacology.

[5] Friedman JR, Webster BM, Mastronarde DN, Verhey KJ, Voeltz GK (2010). ER sliding dynamics and ER-mitochondrial contacts occur on acetylated microtubules. J. Cell Biol..

[6] Guizetti J (2011). Cortical constriction during abscission involves helices of ESCRT-III-dependent filaments. Science.

[7] Jaglin XH, Chelly J (2009). Tubulin-related cortical dysgeneses: microtubule dysfunction underlying neuronal migration defects. Trends Genet..

[8] Kuijpers M, Hoogenraad CC (2011). Centrosomes, microtubules and neuronal development. Mol. Cell. Neurosci..

[9] Begou M (2008). The stop null mice model for schizophrenia displays [corrected] cognitive and social deficits partly alleviated by neuroleptics. Neuroscience.

[10] Fradley RL (2005). STOP knockout and NMDA NR1 hypomorphic mice exhibit deficits in sensorimotor gating. Behav. Brain Res..

[11] Fournet V (2012). The deletion of STOP/MAP6 protein in mice triggers highly altered mood and impaired cognitive performances. J. Neurochem..

[12] Volle J (2013). Reduced expression of STOP/MAP6 in mice leads to cognitive deficits. Schizophr. Bull..

[13] Andrieux A (2002). The suppression of brain cold-stable microtubules in mice induces synaptic defects associated with neuroleptic-sensitive behavioral disorders. Genes Dev..

[14] Powell KJ (2007). Cognitive impairments in the STOP null mouse model of schizophrenia. Behav Neurosci.

[15] Brun P (2005). Dopaminergic transmission in STOP null mice. J. Neurochem..

[16] Bouvrais-Veret C (2007). Sustained increase of alpha7 nicotinic receptors and choline-induced improvement of learning deficit in STOP knock-out mice. Neuropharmacology.

[17] Fournet V (2010). The deletion of the microtubule-associated STOP protein affects the serotonergic mouse brain network. J. Neurochem..

[18] Brenner E, Sonnewald U, Schweitzer A, Andrieux A, Nehlig A (2007). Hypoglutamatergic activity in the STOP knockout mouse: a potential model for chronic untreated schizophrenia. J. Neurosci. Res..

[19] Delotterie D (2010). Chronic administration of atypical antipsychotics improves behavioral and synaptic defects of STOP null mice. Psychopharmacology (Berl)..

[20] Fournet V (2012). Both chronic treatments by epothilone D and fluoxetine increase the short-term memory and differentially alter the mood status of STOP/MAP6 KO mice. J Neurochem.

[21] Deloulme JC (2015). Microtubule-associated protein 6 mediates neuronal connectivity through Semaphorin 3E-dependent signalling for axonal growth. Nat. Commun..

[22] Ishizuka N (2001). Laminar organization of the pyramidal cell layer of the subiculum in the rat. J. Comp. Neurol..

[23] Kishi T (2000). Topographical organization of projections from the subiculum to the hypothalamus in the rat. J. Comp. Neurol..

[24] Namura S, Takada M, Kikuchi H, Mizuno N (1993). Topographical organization of subicular neurons projecting to subcortical regions. Brain Res. Bull..

[25] Swanson LW, Cowan WM (1977). An autoradiographic study of the organization of the efferent connections of the hippocampal formation in the rat. J. Comp. Neurol..

[26] Wright NF, Erichsen JT, Vann SD, O’Mara SM, Aggleton JP (2010). Parallel but separate inputs from limbic cortices to the mammillary bodies and anterior thalamic nuclei in the rat. J. Comp. Neurol..

[27] Porrero C, Rubio-Garrido P, Avendaño C, Clascá F (2010). Mapping of fluorescent protein-expressing neurons and axon pathways in adult and developing Thy1-eYFP-H transgenic mice. Brain Res..

[28] Kerbler GM (2012). Diffusion-weighted magnetic resonance imaging detection of basal forebrain cholinergic degeneration in a mouse model. Neuroimage.

[29] Ren T, Zhang J, Plachez C, Mori S, Richards LJ (2007). Diffusion tensor magnetic resonance imaging and tract-tracing analysis of Probst bundle structure in Netrin1- and DCC-deficient mice. J. Neurosci..

[30] Jiang Y, Johnson GA (2010). Microscopic diffusion tensor imaging of the mouse brain. Neuroimage.

[31] Gimenez U (2016). Microscopic DTI accurately identifies early glioma cell migration: correlation with multimodal imaging in a new glioma stem cell model. NMR Biomed..

[32] Henriksen, R., Johnsson, M., Andersson, L., Jensen, P. & Wright, D. *The domesticated brain: genetics of brain mass and brain structure in an avian species*. *bioRxiv*, doi:10.1101/066977 (2016). 10.1038/srep34031 PMC5043184 27687864

[33] Barton, R. A. In *Evolution of Nervous Systems* **3**, 97–102 (2010).

[34] Couégnas A, Schweitzer A, Andrieux A, Ghandour MS, Boehm N (2007). Expression pattern of STOP lacZ reporter gene in adult and developing mouse brain. J. Neurosci. Res..

[35] Gory-Faure S (2006). STOP-like Protein 21 Is a Novel Member of the STOP Family, Revealing a Golgi Localization of STOP Proteins. J. Biol. Chem..

[36] Nenadic I, Gaser C, Sauer H (2012). Heterogeneity of brain structural variation and the structural imaging endophenotypes in schizophrenia. Neuropsychobiology.

[37] Pergola, G., Selvaggi, P., Trizio, S., Bertolino, A. & Blasi, G. The Role of the Thalamus in Schizophrenia from a Neuroimaging Perspective. *Neurosci. Biobehav.* **54**, 57–75 (2015). 10.1016/j.neubiorev.2015.01.013 25616183

[38] Rasser PE (2010). Cerebellar grey matter deficits in first-episode schizophrenia mapped using cortical pattern matching. Neuroimage.

[39] Walther S (2015). Psychomotor symptoms of schizophrenia map on the cerebral motor circuit. Psychiatry Res..

[40] Phillips JR, Hewedi DH, Eissa AM, Moustafa AA (2015). The cerebellum and psychiatric disorders. Front. public Heal..

[41] Buckner RL (2013). The cerebellum and cognitive function: 25 years of insight from anatomy and neuroimaging. Neuron.

[42] Corcoba A (2016). Glutathione Deficit Affects the Integrity and Function of the Fimbria/Fornix and Anterior Commissure in Mice: Relevance for Schizophrenia. Int. J. Neuropsychopharmacol..

[43] Kulak A, Cuenod M, Do KQ (2012). Behavioral phenotyping of glutathione-deficient mice: Relevance to schizophrenia and bipolar disorder. Behav. Brain Res..

[44] Bopp, M. H. A. *et al*. White matter integrity and symptom dimensions of schizophrenia: A diffusion tensor imaging study. *Schizophr. Res*. **184**, 59–68 (2017). 10.1016/j.schres.2016.11.045 28012640

[45] Asami T (2014). Cerebral white matter abnormalities and their associations with negative but not positive symptoms of schizophrenia. Psychiatry Res. Neuroimaging.

[46] Ćurčić-Blake, B. *et al*. Interaction of language, auditory and memory brain networks in auditory verbal hallucinations. *Prog. Neurobiol.* **148**, 1–20 (2017). 10.1016/j.pneurobio.2016.11.002 PMC5240789 27890810

[47] Douaud G (2007). Anatomically related grey and white matter abnormalities in adolescent-onset schizophrenia. Brain.

[48] White T, Ho B-C, Ward J, O’Leary D, Andreasen NC (2006). Neuropsychological Performance in First-Episode Adolescents with Schizophrenia: A Comparison with First-Episode Adults and Adolescent Control Subjects. Biol. Psychiatry.

[49] Spring S, Lerch JP, Henkelman RM (2007). Sexual dimorphism revealed in the structure of the mouse brain using three-dimensional magnetic resonance imaging. Neuroimage.

[50] Schindelin J (2012). Fiji: an open-source platform for biological-image analysis. Nat. Methods.

[51] Paxinos, G. & Franklin, K. B. J. Paxinos and Franklin’s the Mouse Brain in Stereotaxic Coordinates, 3th Edition. *Elsevier* (2008).

[52] Fillard, P. & Toussaint, N. Medical Image Navigation and Research Tool by INRIA (MedINRIA). *Enseignement* 1–39 (2006).

[53] Susaki, E. A. *et al*. Whole-brain imaging with single-cell resolution using chemical cocktails and computational analysis. *Cell* **157**, 726–39 (2014). 10.1016/j.cell.2014.03.042 24746791

