# Supplementary material for: 3D imaging of the brain morphology and connectivity defects in a model of psychiatric disorders: MAP6-KO mice
Source: Sci Rep. 2017 Sep 4;7:10308. doi: 10.1038/s41598-017-10544-2 (PMC5583184; doi:10.1038/s41598-017-10544-2)
Supplement: Supplementary file 1 — Supplementary Information [file 41598_2017_10544_MOESM1_ESM.pdf]

Supplemental table

3D imaging of the brain morphology and connectivity defects in a model of psychiatric disorders: MAP6-KO mice

Ulysse Gimenez[1,2$], Benoit Boulan[2,3$], Franck Mauconduit[1,2], Fanny Taurel[1,2], Maxime Leclercq[1,2] Eric Denarier[2,3,4], Jacques Brocard[2,3], Sylvie Gory-Fauré[2,3], Annie Andrieux[2,3,4], Hana Lahrech[1,2*], Jean Christophe Deloulme[2,3*]

[1]INSERM, U1205, BrainTech Lab, F-38000, Grenoble, France

[2]Univ. Grenoble Alpes, F-38000, Grenoble, France

[3]INSERM, U1216, Grenoble Institut des Neuroscience, F-38000, Grenoble, France

[4]Commissariat à l'Energie Atomique, BIG-GPC, F-38000, Grenoble, France

$ These authors equally contribute of this study

*These authors equally contribute to this study and equally shared its supervision

***Corresponding authors:** Jean Christophe Deloulme, INSERM, U1216, Grenoble Institute Neuroscience (GIN), F-38000, Grenoble, France; jean-christophe.deloulme@univ-grenoble-alpes.fr & Hana Lahrech, INSERM, U1205, BrainTech Lab, F-38000, Grenoble, France; hana.lahrech@univ-grenoble-alpes.fr

Neuronal tracts	restricting ROIs	excluding ROIs
**anterior commissure (ac)**	S; lateral: -004 mm; ac	H; bregma: -3.60 mm; sm, f and st
**intrabulbar anterior commissure (aci)**	C; bregma: 2.34 mm; aci	S; lateral: -004 mm; ac
	C; bregma: 2.22 mm; aci	C; bregma: 1.10 mm; aca and gcc
**corpus callosum (cc)**	S; lateral: -004 mm; gcc and cc	S; lateral: -004 mm; fr and vhc
		C; bregma: 0.02 mm; f
		C; bregma: 1.18 mm, septal regions
**cerebral peduncle (cp)**	C; bregma: -2.30 mm; cp	C; bregma: -0.94 mm; opt
		S; lateral: -0.04 mm; ac, f, vhc and cc
		C; bregma: -2.46; ec and cc
**fornix system (f)**	C; bregma: 0.02 mm; f	S; lateral -0.04 mm; ac
		C; bregma: 0.14 mm; ac

**fasciculus retroflexus (fr)**	C; bregma: -2.46 mm; fr	C; bregma: -0.82; sm
**internal capsule (ic)**	C; bregma: -1.06 mm; ic	S; lateral -004 mm; cc
		C; bregma: -2.46 mm; ec and cc
**mammillary tract (mt)**	C; begma: -1.34 mm; mt	
**optic tract (opt)**	C; bregma: 2.74 mm; opt	C; bregma: -1.34 mm; fi
		C; bregma: -0.82 mm; vhc and fi
		H; bregma: -4.12 mm; VDB and Nv
**pyramidal tract (py)**	C; bregma: -5.88; pyr	
**stria medularis (sm)**	C; bregma: -0.34 mm; sm	C; bregma: 0.02 mm; f and aca
		S; lateral: 0.96 mm; fi
		H; bregma: -3.28 mm; st, ic, and f
**stria terminalis (st)**	S; lateral: 0.96 mm; st	C, bregma: -2.46 mm; cc, cg

		and ec
		C; bregma: -0.22 mm; ds, vhc and Sfi

Supplementary Table 1: Positions of restricting and excluding ROIs used to reconstruct individual neuronal tracts. Positions are indicated in line with the coordinates published in the Paxinos mouse atlas [2]. C, S and H indicate the coronal, sagittal and horizontal planes, respectively. Abbreviations of neuronal tract or brain regions indicate positions of ROIs.

Legend Movie 1: The movie shows a sagittal view of the fornix system reconstructed from middle-hemisphere of WT/Thy-eYFP-H (left panel) and KO/Thy-eYFP-H (right panel) using tissue –clearing method. Related to the figure 2
